# Spinal Stenosis: An Emerging Complication of Ageing in People With Haemophilia

**DOI:** 10.1111/hae.70089

**Published:** 2025-07-29

**Authors:** Claire Kelly, Mark McGowan, Niamh Larkin, Jake M. Mc Donnell, Anne‐Marije Hilshof, Mary Byrne, Catherine Bergin, Aine O'Gara, Kevin Ryan, Mairead O'Donovan, Niamh O'Connell, Keith Synnott, Joseph S. Butler, Stacey Darwish, Brian O'Mahony, Megan Kennedy, Peter L. Turecek, James S. O'Donnell, John Gormley, Michelle Lavin

**Affiliations:** ^1^ National Coagulation Centre St. James's Hospital Dublin Ireland; ^2^ Irish Centre for Vascular Biology School of Pharmacy & Biomedical Sciences RCSI Dublin Ireland; ^3^ Department of Physiotherapy St. James's Hospital Dublin Ireland; ^4^ National Spinal Injuries Unit Department of Trauma and Orthopaedic Surgery Mater Misericordiae University Hospital Dublin Ireland; ^5^ Royal College of Surgeons Dublin Ireland; ^6^ Department of Anaesthetics St. James's Hospital Dublin Ireland; ^7^ School of Medicine University College Dublin Dublin Ireland; ^8^ Irish Haemophilia Society Cathedral Court Dublin Ireland; ^9^ Discipline of Physiotherapy School of Medicine Trinity College Dublin the University of Dublin Dublin Ireland; ^10^ Baxalta Innovations GmbH a Takeda Company Vienna Austria

**Keywords:** ageing, arthropathy, haemophilia, spinal stenosis, spine, surgery

## Abstract

**Introduction:**

Advances in haemophilia care have brought the challenges of ageing for people with haemophilia (PWH) to the forefront. Age‐related spinal degeneration may result in spinal stenosis; however, the rates in PWH are unknown. We sought to systematically review Irish PWH to address this gap in the current literature.

**Methods:**

Clinical and radiological notes of all patients ≥40 years old (yo) registered with severe or moderate haemophilia A or B were reviewed, recording Haemophilia Joint Health Scores (HJHS), radiological imaging and orthopaedic/pain interventions.

**Results:**

Of 100 males included with moderate or severe haemophilia, 13% had radiologically confirmed symptomatic spinal stenosis (reported rates 4% in the general population aged >60 yo). Persons with stenosis were older (median age 69yo vs. 55 yo, *p* = 0.004) with similar rates observed between those with moderate and severe haemophilia (4/35, 11.4% vs. 9/65, 13.8%). HJHS did not differ between those with and without stenosis (median 30 vs. 35, *p* = 0.6). On regression analysis, only age >60 yo was associated with an increased likelihood of spinal stenosis; severity of haemophilia (moderate vs. severe) was not significantly associated.

**Conclusions:**

These data identify symptomatic spinal stenosis as a novel complication of ageing for PWH. Spinal stenosis rates were higher than expected for age in comparison to reported rates in the general population. Current joint assessments fail to capture spinal pathology, highlighting limitations of HJHS in older PWH. Increased awareness amongst PWH and health care providers of spinal stenosis is directly required; however, optimal management strategies for PWH with established stenosis are yet to be defined.

## 1 Introduction

Therapeutic advances in the last decade have dramatically altered the lived experience and prognosis for people with haemophilia (PWH), especially those with severe disease. Extended half‐life products, novel therapies and gene therapy offer real opportunities for a bleed‐free life in those who can access them. In combination with highly active antiretroviral (HAART) and hepatitis C eradication therapies, the life expectancy of PWH is, for the first time, similar to that of the general population [1]. With these marked improvements in care, we are now faced with new challenges arising from age‐related complications in older PWH. While novel therapies have induced major improvements in bleeding rates in PWH, they will not reverse the decades of accumulated joint damage, which can continue to restrict mobility and impair quality of life in older PWH [2, 3, 4]. As a result, multi‐joint extensive arthropathy is common in older PWH, complicating the general health comorbidities experienced with age [1]. With this newly ageing cohort, there may also be additional, unforeseen complications due to the sequelae of long‐term arthropathy and altered physical activity [5]. These new clinical complications require alterations and adaptations to our current assessment pathways, which utilise tools such as the Haemophilia Joint Health Score (HJHS) derived from paediatric haemophilia populations. The applicability of this score in the global assessment of physical and musculoskeletal health in older PWH is questionable, as it may overlook additional or new age‐related musculoskeletal changes that may occur in PWH [6].

**FIGURE 1:**
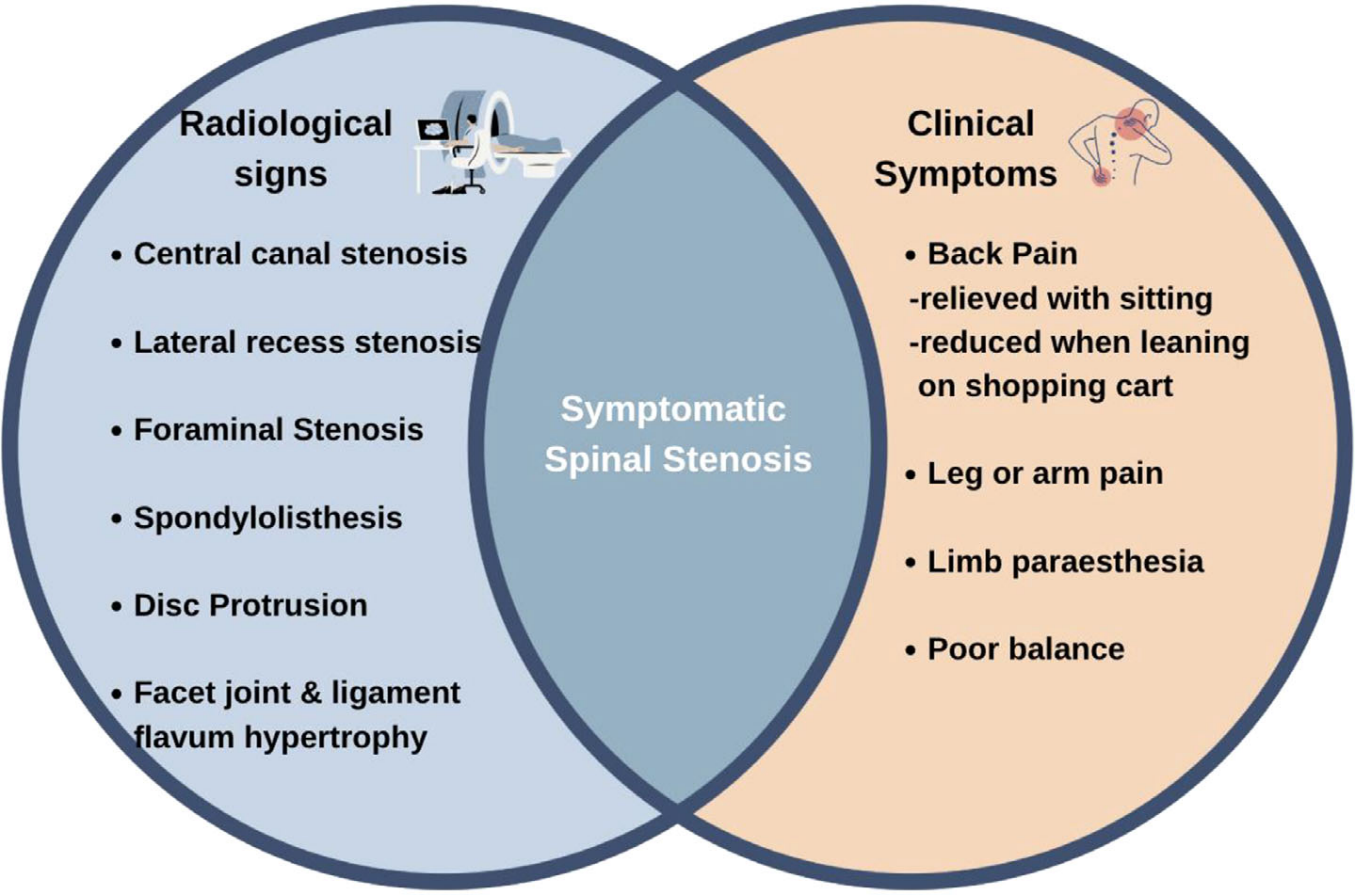
Radiological changes of spinal stenosis or symptoms of back pain may exist in isolation, symptomatic spinal stenosis requires the presence of both.

Lower back pain is common with aging in the general population. Extensive degeneration of the intervertebral discs, ligamentum flavum and facet joints can result in spinal stenosis—narrowing of the spinal perineurovascular space resulting in compression of the central canal and/or exiting nerves [7, 8]. Symptomatic spinal stenosis is characterized by leg/buttock pain, motor or sensory disturbances while walking, with relief noted on forward flexion (typically reported when bending over using a shopping cart or bicycle). Bilateral lower extremity weakness or pain may occur with a negative straight leg test but a positive 30‐s extension test and preservation of pulses [9, 10]. The prevalence of spinal stenosis in the general population increases with age but varies depending on the criteria used. Radiological changes of spinal stenosis may be seen in asymptomatic individuals, impacting approximately 20% of those aged ≥60 years old (yo) [11]. Intervention is not required unless symptomatic spinal stenosis is present, characterised by typical clinical signs of stenosis in combination with radiological changes [12, 13] (Figure 1). Symptomatic spinal stenosis also increases with age but occurs less frequently, with reported rates of approximately 4% in those ≥60 yo [12].

During the follow‐up of our Irish cohort of PWH, we noted increased physiotherapy input for spinal stenosis. Given the lack of data on spinal stenosis in older PWH, we sought to systematically review all older adults (≥40 yo) with moderate and severe haemophilia attending the Irish National Coagulation Centre (NCC) to determine the overall rates of spinal stenosis and whether this exceeded that expected in the general population. We also sought to understand the relationship, if any, between pre‐existing arthropathy and spinal stenosis and to identify potential risk factors in PWH for the development of spinal stenosis.

## 2 Methods

### 2.1 Study Design

Ethical approval for the Irish Personalised Approach to the Treatment of Haemophilia (iPATH) study and secondary audit was granted by the St. James's Hospital and Tallaght University Hospital Research Ethics Committee (reference number 4044). Recruitment via the iPATH programme occurred as previously described [2]. The clinical records of all persons with moderate (baseline factor VIII/IX 1–5 IU/dL) or severe (baseline factor VIII/IX <1 IU/dL) haemophilia aged ≥40 yo registered with the NCC were systematically reviewed. Patients with congenital spinal stenosis or those not under long‐term follow‐up (e.g., registered while temporarily in Ireland) were excluded. All collected data were stored, coded and pseudonymized in line with data protection regulations.

#### 2.1.1 Clinical and Radiological Data

Orthopaedic surgeries, joint injections/procedures and HJHS were recorded for each individual.  The International Prophylaxis Study Group (IPSG) HJHS version 2.1 is currently utilised in our centre [6, 14]. The domains reviewed include swelling, duration of swelling, muscle atrophy, crepitus, flexion loss, extension loss, pain, strength and global gait score. All HJHS over the preceding 15 years were performed by one of three specialist physiotherapists based in the NCC, minimizing variability. Body mass index (BMI) was obtained from the most recent clinical review, and the outcome and date of the most recent bone densitometry (DXA) were documented where available.

The reports of all radiological imaging (x‐ray [XR], computerized tomography [CT], magnetic resonance imaging [MRI], ultrasound [US]) performed from 2003–2023 were reviewed. All imaging was performed due to symptoms of concern, as routine radiological screening in asymptomatic patients is not undertaken in our centre. For each test, the data modality, indication and outcome were recorded. Clinical records were interrogated to determine the indication for all imaging (i.e., back pain, limb radiation, pain exacerbated by movement and relieved by bending forward) as well as for spontaneous or traumatic spinal bleeds. The diagnosis of spinal stenosis relied on MRI findings, and each MRI was retrospectively reviewed for this study to ensure fidelity of diagnosis and ensure consistency of classification using the Lurie grading system [15]. From this comprehensive review, all PWH with both radiological changes and symptoms consistent with spinal stenosis were identified; referred to as ‘symptomatic spinal stenosis’ throughout this paper. Bone DXA scans measured T‐scores and *Z*‐scores at three anatomical sites (the lumbar spine [L1‐L4], the femoral neck and hip) as well as a vertebral fracture assessment on the lumbar and thoracic spine where feasible. DXA results were graded as per the World Health Organisation (WHO) classification [16].

### 2.2 Data Analysis

Data analysis was performed on GraphPad Prism Version 10.4.1 with comparative analysis using paired and unpaired *t*‐tests or Mann–Whitney test for non‐parametric analysis as appropriate. To further address the association between potential clinical determinants and a diagnosis of spinal stenosis in PWH, univariable logistic regression analysis was performed using the R statistical software packages ‘stats’ and ‘forestplot’. Results are presented in a Forest plot, illustrating the odds ratios along with their corresponding 95% confidence intervals. A *p* value < 0.05 was considered statistically significant.

## 3 Results

### 3.1 Demographics

All PWH aged ≥40 yo with moderate or severe haemophilia registered to our centre were identified (*n* = 108). Those with known congenital spinal stenosis (*n* = 2) and those who were not under long‐term follow‐up (*n* = 6) were excluded. The remaining 100 (100% male) had an in‐depth review.

**FIGURE 2:**
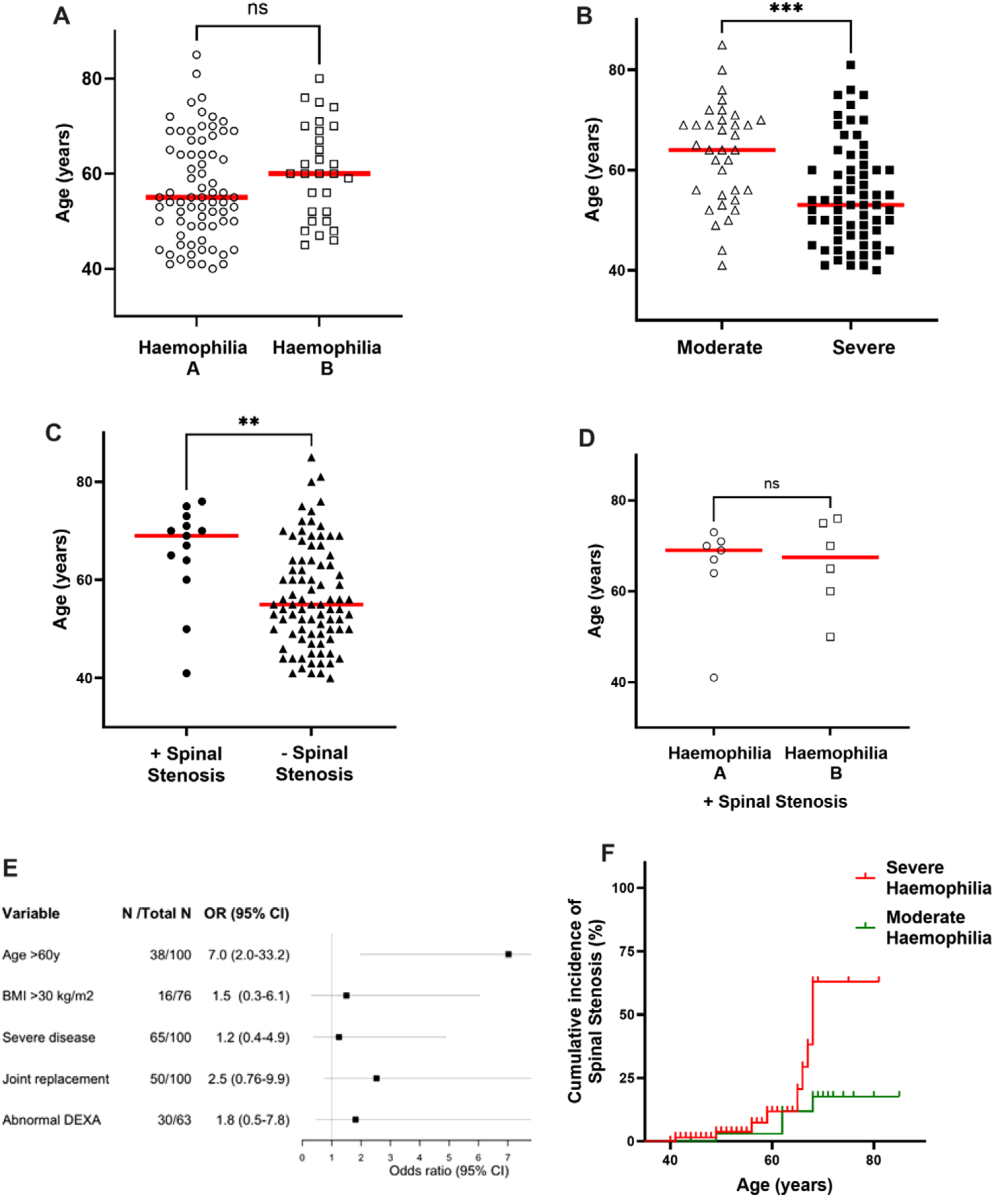
Scatter plot (Figure 2A) comparing ages of those with haemophilia A (circle, median 55 years old, yo) and haemophilia B (squares, median 60yo, *p* = 0.1). People with moderate haemophilia (Figure 2B, triangles) were significantly older (median 64yo) than those with severe haemophilia (squares, median 53 yo, *p* = 0.0002). Overall, the age profile of those with spinal stenosis (Figure 2C, squares) was older (median 69 vs. 55 yo, *p* = 0.004). For those with spinal stenosis, the age profile was similar between those with haemophilia A (Figure 2D, circles, median 69yo) and haemophilia B (open squares, median 67.5yo, *p* = 0.9). Figure 2E displays a Forest plot of clinical determinants, including age, body mass index (BMI), severe haemophilia, prior joint replacement, and abnormal DEXA scan, in relation to a diagnosis of spinal stenosis. Squares represent the odds ratios (OR), and whiskers indicate the corresponding 95% confidence intervals. Only age was associated with an increased likelihood of a spinal stenosis diagnosis following logistic regression analysis. Figure 2F shows a Kaplan–Meier curve assessing the cumulative incidence of spinal stenosis development for those with moderate (green line) or severe haemophilia (red).

Of the 100 PWH, 71% had haemophilia A and 29% had haemophilia B, with moderate haemophilia in 35 (35%) and severe in 65 (65%) (Table 1). The median age of the group overall was 56 yo (range 40—85 yo), with no significant difference in age between those with Haemophilia A or B (median 55 vs. 60 yo, *p* = 0.1, Figure 2A). Of note, the age profile of those included with moderate haemophilia was significantly older compared to those with severe haemophilia overall (median 64 vs. 53 yo, *p* = 0.0002, Figure 2B).

**TABLE 1:** Demographic information of included patients.

	Total (*n* = 100)	Spinal stenosis (*n* = 13)	No spinal stenosis (*n* = 87)
Age years [median,(range)]	56 (40–85)	69 (41–76)	55 (40–85)
Severe haemophilia A	46	4	42
Moderate haemophilia A	25	3	22
Severe haemophilia B	19	5	14
Moderate haemophilia B	10	1	9
Inhibitors (*n*)	14	2	12
Current	5	1	4
Historical	9	1	8
Bethesda assay			
>5 Bu	5	0	5
<5 Bu	9	2	7
Treatment regimen *n* (%)			
Prophylaxis	69 (69%)	9 (69%)	60 (69%)
On demand	31 (31%)	4 (31%)	27 (31%)
HJHS [median,(range)]	(*n* = 70)	(*n* = 10)	(*n* = 60)
Total	35 (4–78)	30 (20–50)	4 (35–78)
Left ankle	6 (0–14)	5.5 (1–9)	6 (0–14)
Right ankle	6.5 (0–15)	6 (2–9)	7 (0–15)
Left knee	3 (0–15)	2.5 (0–10)	3 (0–15)
Right knee	3 (0–13)	2.5 (0–13)	3 (0–13)
Left elbow	5 (0–15)	1.5 (0–9)	6 (0–15)
Right elbow	5 (0–18)	6 (0–12)	5 (0–18)
Global gait score	4 (2–4)	4 (4–4)	4 (2–4)
BMI	(*n* = 76)	(*n* = 11)	(*n* = 65)
Median (range)	27.2 (20.3–42.5)	28.2 (21.4–42.5)	26.9 (20.3–41.5)
% Normal weight	25%	9%	28%
% Overweight	54%	64%	52%
% Obese	21%	27%	20%
Bone densitometry	(*n* = 63)	(*n* = 10)	(*n* = 53)
Normal	33 (52%)	4 (40%)	29 (55%)
Osteopenia	23 (37%)	4 (40%)	19 (36%)
Osteoporosis	7 (11%)	2 (20%)	5 (9%)

Abbreviations: BMI, body mass index; DXA, dual‐energy x‐ray absorptiometry; HJHS, Haemophilia Joint Health Score.

### 3.2 Prevalence of Spinal Stenosis in Our Cohort of PWH

In this group of 100 older PWH, 40% (40/100) had spinal radiology performed, with 32% (32/100) undergoing MRI imaging, due to reported back pain and/or neurological symptoms. From this, 13% (13/100) of PWH had a diagnosis of symptomatic spinal stenosis confirmed due to the presence of both radiological changes and consistent clinical symptoms. Following a review of all MRI imaging, all diagnoses of spinal stenosis were independently confirmed, with no changes in diagnoses seen.

### 3.3 Rates of Spinal Stenosis by Type of Haemophilia

Although haemophilia B is often perceived as having a ‘milder’ phenotype, rates of stenosis were higher in this group, with 20.7% (6/29) of men with haemophilia B affected versus 9.8% (7/71) of those with haemophilia A, despite a similar age profile [17, 18]. Comparable rates were also seen between those with moderate (4/35, 11.4%) and severe haemophilia (9/65, 13.8%) (Table 1), although subgroup analysis was limited by the number of patients involved. No difference in rates of inhibitor development was seen between those with and without spinal stenosis (2/13 [15.4%] vs. 12/87 [13.8%], Table 1). As all PWH reviewed were aged ≥40 yo, none had access to early prophylaxis, with tertiary prophylaxis (after the establishment of arthropathy, *n* = 69, 69%) or on‐demand treatment (*n* = 31, 31%, all moderate haemophilia) employed. Rates of transfusion‐associated infections (TTI) were also similar between those with or without stenosis (exposure to Hepatitis B 38% vs. 46%, past Hepatitis C infection 92 % vs. 79% or HIV 15% vs. 28%, respectively). All patients with HIV were on HAART, and all those with exposure to Hepatitis B and C had cleared infection spontaneously or following eradication therapy.

### 3.4 Influence of Age on the Development of Spinal Stenosis

The subgroup with spinal stenosis were significantly older than those without (median age 69vs. 55 years, *p* = 0.004, Figure 2C). Overall, only 3.5% (2/57) of people aged 40–59 years old had spinal stenosis; however, this rose sharply thereafter, with 19.2% (5/26) of those aged 60–69 yo and 35.3% (6/17) of those ≥70 years affected. Severity of haemophilia varied amongst those with spinal stenosis, four with moderate haemophilia and nine with severe haemophilia. The median age at diagnosis with spinal stenosis was not altered by haemophilia type (haemophilia A, *n* = 7, 69 years vs. haemophilia B, *n* = 6, 68 years; *p* = 0.9, Figure 2D) or severity (moderate, *n* = 4, 66 years vs. severe, *n* = 9, 70 years, *p* = 0.3). Regression analysis was performed to examine clinical determinants including age, BMI, severity of haemophilia, prior joint replacement and abnormal DEXA scan, in relation to a diagnosis of spinal stenosis. Only age was associated with an increased likelihood of a spinal stenosis diagnosis following logistic regression analysis (Figure 2E). To assess the risk of developing spinal stenosis over time for those with moderate or severe haemophilia, a cumulative incidence curve was generated, highlighting the divergence over time with higher cumulative incidence in those with severe haemophilia (Figure 2F).

### 3.5 Both Lumbar and Extralumbar Spinal Stenosis Were Identified in PWH

Although the lumbar spine was most commonly involved (9/13 cases), stenosis was identified in the cervical (*n* = 5/13, 38%) and thoracic (*n* = 1/13, 8%) spine; two patients had involvement of more than one spinal region (one with cervical and lumbar stenosis, one with thoracolumbar stenosis) (Table 2). No patient had previously reported spontaneous or traumatic bleeding to these sites. Neurogenic claudication was reported in all cases of lumbar spinal stenosis. Additionally, upper extremity radicular symptoms were reported in two patients with concurrent cervical stenosis. Lumbar stenosis was graded as mild‐moderate in the majority of cases (*n* = 7) and moderate in the remaining two cases. For those with cervical stenosis, both cases were denoted as mild, while the case of thoracolumbar stenosis was reported as moderate. XR imaging showed reduced lumbar flexion‐extension in three cases, with additional dynamic stenosis in three cases.

**TABLE 2:** Case series of persons with spinal stenosis (*n* = 13) including background diagnosis, radiological findings and joint status.

	Case 1	Case 2	Case 3	Case 4	Case 5	Case 6	Case 7	Case 8	Case 9	Case 10	Case 11	Case 12	Case 13
Haemophilia													
Disease severity and type	SHB	SHB	SHA	SHA	SHA	SHB	MHA	MHA	SHB	MHA	MHB	SHB	SHA
Current Age	76	75	73	71	70	70	69	67	65	64	60	50	41
Current HJHS	37	46	50	N/A	21	42	32	20	21	24	N/A	28	37
Current BMI	27.30	28.72	21.37	N/A	28.17	25.29	34.38	42.52	28.43	26.89	N/A	27.17	35.14
DXA Age at DXA	Osteopenia 70	Osteopenia 69	Osteoporosis 67	N/A	Normal 66	Osteoporosis 63	Normal 65	Normal 63	Osteopenia 60	Osteopenia 62	N/A	Normal 44	N/A
Spinal stenosis													
Age at diagnosis	68	56	67	59	68	66	68	62	65	62	49	49	41
Site	Lumbar L1‐L2	Lumbar L4/L5 L5/S1	Lumbar L3/L4 L4/L5	Lumbar L3/L4	Lumbar L4/L5 L5/S1	Lumbar L1/L2 L3/L4	Thoraco‐lumbar T10‐T11 L4‐L5 L5‐S1	Lumbar L3/L4 L4/L5	Cervical Lumbar C3/C4 C4/C5 L4/L5	Cervical C4/C5	Cervical C6/C7	Cervical C3/C4 C5/C6 C6/C7	Cervical C4/C5 C5/C6
Severity (if available)	Moderate	NA	Mild	Mild	Moderate	Severe	Moderate	NA	NA	Mild	NA	Mild	Mild‐Mod
Type of MRI	T1& T2	T1& T2	T1& T2	T1 & T2	T1 & T2	T1 & T2	T1 & T2	T1 & T2	T1 & T2	T2	T1 & T2	T1 & T2	T2
Arthropathy													
Lower limb joints replaced or fused	Lt knee	B/L ankle B/L knee	B/L knee, Lt ankle	B/L knee	Rt ankle Rt knee	Left knee	0	0	Rt ankle, Lt knee	Lt ankle	0	B/L ankle	0
Age @ 1st joint operation	62	52	53	63	45	56	—	—	46	61	—	47	0
Joint injections	B/L ankle	B/L ankle	Lt ankle	0	0	B/L ankle spinal	Rt knee Rt hip	0	Spinal	0	0	Spinal B/L ankle	0
No. of injections	2	4	3	0	0	5	2	0	3	0	0	14	0
Spinal rhizotomy	No	No	No	No	No	Yes	Yes	No	Yes	No	No	No	No

Abbreviations: B/L, bilateral; BMI, body mass index; DXA, dual‐energy x‐ray absorptiometry; HJHS, Haemophilia Joint Health Score; Lt, left; N/A, not available; Rt, right.

### 3.6 HJHS Did Not Differ Between Those With and Without Spinal Stenosis

**FIGURE 3:**
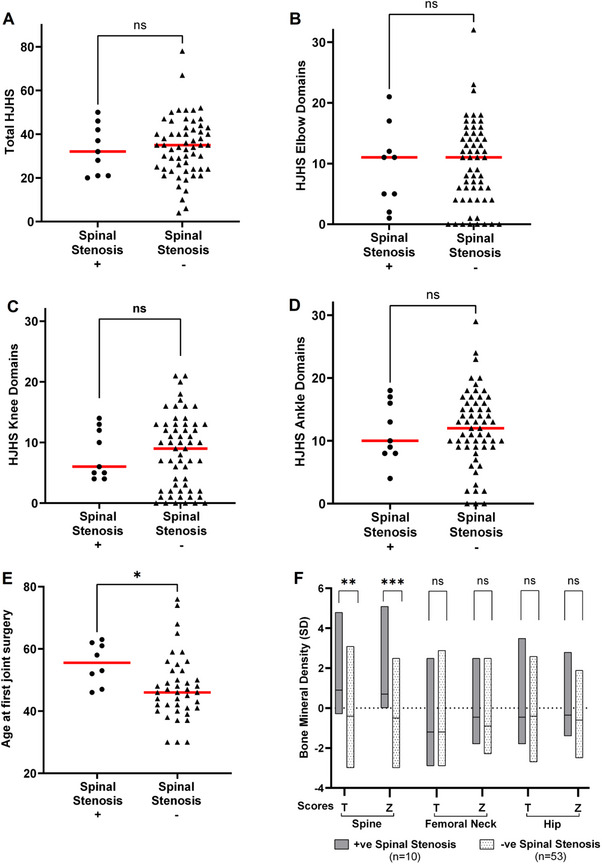
Scatter plots showing baseline arthropathy as assessed by the HJHS were similar between those with (Figure 3A, circles, median 30) and without spinal stenosis (triangles, median 35, *p* = 0.55). When those with (circles) and without (triangles) spinal stenosis were compared, no significant differences were seen in HJHS scores for elbows (Figure 3B median 8 vs. 11, *p* = 0.62), knees (Figure 3C median 5.5 vs. 9, *p* = 0.83) or ankles (Figure 3D median 11.5 vs. 12, *p* = 0.83). The age at first joint surgery was significantly higher in those with SS (median 55.5yo vs. 46yo, *p* = 0.02, Figure 3E). Figure 3F: Box chart DXA bone mineral density (BMD) scores for those with (*n* = 10) and without (*n* = 53) spinal stenosis. T and Z scores are compared by location; spine (T 0.9 vs. −0.4, *p* = 0.007; Z 0.7 vs. −0.5, *p* = 0.0007), femoral neck (T 1.2 vs. 1.2, *p* = 0.942; Z −0.45 vs. −0.9, *p* = 0.255) and hip (T 0.45 vs. 0.4, *p* = 0.600; Z −0.35 vs. −0.6, *p* = 0.26).

The HJHS remains the standard of care for assessment of arthropathy in haemophilia. As this focuses on large joint arthropathy and impairments of gait, we assessed the ability of the HJHS to differentiate between those with and without spinal stenosis. For each individual, overall and subdomain HJHS results were analysed where available (*n* = 70). Total HJHS were similar between those with or without spinal stenosis (median 30 vs. 35, *p* = 0.6, Figure 3A) and no significant difference was identified between combined elbow (median 8 vs. 11, *p* = 0.6), knee (median 5.5 vs. 9, *p* = 0.8) or ankle scores (median 11.5 vs. 12, *p* = 0.8) (Figure 3B–D). Of note, the global gait score was particularly limited in the assessment of older PWH as this domain quickly saturated, with the maximum domain score (4/4) seen in 97% of those aged ≥40 years. When HJHS over time were reviewed, the total HJHS at time of diagnosis with spinal stenosis did not significantly alter from HJHS 5 years prior (median 38 vs. 34, *p* = 0.2, *n* = 7). Multi‐joint arthropathy (joints with HJHS domain score >0) was more common in those with stenosis (only 1/13 with ≥2 joints domain score of 0) in comparison to 18 (30%) of people without spinal stenosis.

### 3.7 Age at First Joint Replacement Was Significantly Older in Those With Spinal Stenosis

The total number of joint replacements or ankle fusions was similar between those with and without spinal stenosis (*n* = 49; median 2 vs. 1, *p* = 0.18). The only arthropathy‐related variable found to differ was the age at first joint replacement/ankle fusion, which was markedly older in those who subsequently developed spinal stenosis (median 55.5 vs. 46 years, *p* = 0.02, 3E). On review of clinical notes, in many of these instances, patients had elected to delay joint replacement following discussion.

### 3.8 Osteopenia and Osteoporosis Were More Common in Those With Spinal Stenosis

Reduced physical activity can have negative health impacts on BMI, as well as on cardiovascular and bone health. Using available recent BMI (*n* = 76) and DXA (*n* = 63) data, we reviewed the rates of obesity, osteopenia and osteoporosis in our cohort. Data were not available on all patients due to the delays and difficulties accessing routine tests and in‐person assessment during the COVID pandemic. Rates of obesity were marginally increased amongst those with spinal stenosis (27% vs. 20% in those without spinal stenosis) but similar to the general Irish population aged ≥40 years old (27%) [19]. DXA data were available for only 63% of our cohort, 10 persons with spinal stenosis and 53 without. Where assessed, bone mineral density (BMD) profiles differed between those with and without spinal stenosis, with normal BMD in only 40% of those with spinal stenosis compared to 55% of those without stenosis. T and *Z*‐scores were similar between groups, except in the spine, where the spinal stenosis group had surprisingly significantly higher scores (Figure 3F). Higher rates of both osteopenia overall (40% vs. 36%) and osteoporosis (20% vs. 9%) were seen in the spinal stenosis cohort compared to those without stenosis.

### 3.9 Surgical Intervention for Spinal Stenosis Was Not Performed on Affected Patients With Moderate or Severe Haemophilia

Of those with haemophilia and spinal stenosis, none to date have undergone spinal surgical intervention. In some cases, this was due to improvement of symptoms with physiotherapy and pain management prior to surgical review. Two patients had additional significant co‐morbidities precluding consideration of surgical options. In all cases, the risk of bleeding was considered and, in one instance, the diagnosis of haemophilia was specifically listed as a key factor in not proceeding with surgery.

All PWH with spinal stenosis have been managed via a combination of physiotherapy, haemophilia, orthopaedic and/or spinal surgical and pain team reviews. Each patient had an extensive physiotherapy support programme and analgesia. While oral analgesia was used for all patients, 7/13 also required targeted pain injections (e.g., epidural or cervical nerve block) to help manage pain; repeated injections were often required.

## 4 Discussion

Novel therapies have significantly reduced the burden of bleeding in PWH, but the burden of arthropathy remains significant in adults as they age. In this study, we outline the first report of spinal stenosis in older PWH, highlighting rates exceeding those reported in the general population [8]. Awareness, management and most importantly prevention of this condition is essential in a group of patients for whom surgical options may be more limited and who already experience significant pre‐existing musculoskeletal morbidity.

The focus of studies of arthropathy in PWH has traditionally focused on the large joints, with few studies centred on spinal symptomatology and function. Reports of spinal stenosis in PWH have been limited to two case reports [20, 21] and one case series of five men with haemophilia with lumbar spinal stenosis and spondylolisthesis [22]. Studies reporting spinal outcomes in PWH have largely focused on non‐specific lower back pain, which is also highly prevalent in the general population [23, 24]. As symptomatic spinal stenosis is a disease of ageing, predominantly affecting those aged ≥60 yo, it was surprising to see this relatively younger cohort affected. In our study of PWH ≥40 yo, we identified an overall rate of 13% of symptomatic, radiologically proven spinal stenosis. For those aged ≥60 yo, rates of stenosis were considerably higher than expected in the general population, with 18.5% (5/27) of those aged 60—69 yo and 35.3% (6/17) of those ≥70 yo affected [12]. In addition, analysis showed a clear divergence over time in the cumulative incidence between those with moderate and severe haemophilia, although the severity of haemophilia was not significant on regression analysis (Figure 2E). These data should raise awareness and prompt consideration of spinal stenosis in all older adults with haemophilia presenting with back pain.

Routine spinal investigations were not routinely performed in our comprehensive care centre, and therefore, all cases were diagnosed due to investigations prompted by patients’ symptoms, in keeping with the clinical pathways for the general population. Rates of spinal stenosis were similar amongst those with moderate (4/35, 11.4%) and severe haemophilia (9/65, 13.8%) and were higher in those with haemophilia B (20.7%) than haemophilia A (9.8%) despite a similar overall age profile, albeit that subgroup analyses in this paper are limited by sample size. The presence or history of inhibitors or TTI did not increase the risk of spinal stenosis in our cohort. The relatively high rate amongst those with moderate haemophilia may be related to the older age of this group; however, given that access to prophylaxis has often been limited for this group, this may be a contributory factor.

The rates of elevated BMI were of concern in the overall cohort but reflect the general Irish population [19]. Limitations in physical activity due to musculoskeletal morbidity can negatively impact physical fitness, BMI and cardiovascular health. These are particularly important sequalae in a patient cohort who may additionally have significant co‐morbidities such as HIV, hepatitis or hepatic dysfunction, as well as exposure to increased risk of bleeding with the use of antiplatelets or anticoagulation. While bone health was examined in this cohort, limited conclusions can be drawn at this time on the impact of spinal stenosis on bone health as not all PWH had DXA data available.

Given that current physiotherapy assessment tools for PWH are focused on the large joints, we examined their ability to detect spinal disease. We found a high burden of multi‐joint arthropathy in our population. HJHS scores were similar, however, between those with and without spinal stenosis, with no significant difference in elbow, knee or ankle joints seen between groups. The global gait score proved to be of particularly limited benefit in the assessment of adults, as it was already saturated in 97% of adults aged ≥40 yo.

The small size of this cohort precludes a thorough investigation of the underlying causes or contributory factors to the development of spinal stenosis. Haemophilic arthropathy has been shown to result in altered spinal and pelvic postures during gait amongst PWH compared to controls, specifically with respect to higher trunk inclination, lower apex lumbar lordosis and increased pelvic torsion [24]. As adults with multi‐joint arthropathy, many patients had undergone joint replacements, fusions and joint injections for management of arthropathy and pain. The spinal stenosis group were significantly older at the time of first joint replacement/fusion. This raises the possibility that biomechanical alterations due to arthropathy and chronic pain may result in compensatory postures, potentially increasing the risk of degenerative spinal changes. Spinal stenosis is a disease of ageing, and the earlier age at diagnosis may be reflective of accelerated overall ageing, which can occur in PWH, as identified in a recent study identifying altered telomeric structure in PWH [25]. Although major bleeding did not appear to be a contributory factor, there is also the possibility that micro‐bleeds may occur in PWH and contribute to degenerative changes. Clearly, larger studies are required to elucidate the mechanism of spinal stenosis in PWH further.

There is no evidence to guide optimal treatment approaches for PWH who develop spinal stenosis, with only limited case reports available [22, 26]. Surgical options may be more limited for PWH due to concerns about treatment, monitoring and increased bleeding risk. For PWH undergoing spinal surgery, ensuring adequacy of replacement therapy and close monitoring of both clinical status and coagulation factor levels in the perioperative period are paramount. HTCs and spinal services may not be co‐located, hindering direct access to rapid laboratory testing or specialist haemostasis clinical review. New challenges require adaptations to our approaches for care; in our centre, we have responded by developing a multidisciplinary clinical service including a Physiotherapist, the Haemophilia team and an Anaesthetist Pain Specialist. In this way, we aim to optimise medical and physiotherapy approaches, offer rapid access to epidural injections if deemed of benefit and rapidly triage those who require further spinal team input.

We acknowledge that, given the retrospective nature of this study, there are some unavoidable limitations. Whilst our entire registered national cohort of 100 PWH is a reasonable sample size, analysis is limited by the small subgroup size, limiting our ability to explore causal and predictive factors. As a retrospective clinical review, some data sets (e.g., DXA scans) are not available for all PWH, reflecting the real world challenges in service delivery. Recruitment of age‐matched local control groups for longitudinal follow‐up is required to provide a comparator group for analysis. In addition, similar analyses should be taken in other, larger HTCs to validate this work and help increase understanding. Despite these limitations, this work serves to provide the first comprehensive assessment of spinal stenosis in a cohort of well‐phenotyped older PWH. We hope that data generated in other HTCs may add to this work and provide further insights in future, paving the way for larger, multicentre studies.

## 5 Conclusions

As a healthcare community, we need to evolve to the emerging challenges presented by our ageing haemophilia patients. Our traditional approaches to arthropathy remain largely peripheral joint‐based and were developed from studies of children and adolescents with haemophilia. We must recognize that our current pathways may fail to detect spinal stenosis in ageing PWH. As surgical options may be more limited due to bleeding concerns, there is an important need to understand the pathophysiology of spinal stenosis in PWH in order to prevent or mitigate its impact with ageing. The effectiveness of physiotherapy interventions, exercise therapy and/or spinal injections has not been studied to date in PWH and also warrants urgent attention in future research. It is now time to alter our processes, adapt our concepts and meet the needs of our first generation of ageing PWH [27, 28].

## Author Contributions

Claire Kelly, Mark McGowan, Niamh Larkin, Megan Kennedy, Jake M. Mc Donnell and Michelle Lavin collected the data. Claire Kelly, Megan Kennedy, Catherine Bergin, Jake M. Mc Donnell, Anne‐Marije Hilshof, John Gormley, Michelle Lavin analysed the data. All authors Claire Kelly, Mark McGowan, Niamh Larkin, Jake M. Mc Donnell, Anne Marije Hilshof, Mary Byrne, Catherine Bergin, Aine O'Gara, Kevin Ryan, Mairead O'Donovan, Niamh O'Connell, Keith Synnott, Joseph S. Butler, Stacey Darwish, Brian O'Mahony, Megan Kennedy, Peter L. Turecek, James S. O'Donnell, John Gormley, Michelle Lavin) were involved in writing and reviewing the paper. We can confirm there was no use of generative AI (e.g., ChatGPT) in this document.

## Ethics Statement

This retrospective review was conducted in accordance with the principles of the Declaration of Helsinki. Ethical approval for the iPATH study and secondary audit was granted by the St. James's Hospital and Tallaght University Hospital Research Ethics Committee (reference numbers 2017/01/06 and 4044).

## Conflicts of Interest

C.K., J.M., A.M.H., C.B., A.O'G., K.R., M.O'D, K.S., J.S.B., S.D., M.K., have no COIs to declare. M.M. has received speaker fees from Sobi. N.L. has received speaker fees from Sobi. M.B. has served as a consultant for Sobi. N.M.O'C has received financial support for research from SOBI, consultancy fees from F. Hoffmann‐La Roche Ltd, UniQure, SOBI and CSL Behring, and is a member of the Speakers Bureau for F. Hoffmann‐La Roche Ltd, SOBI, CSL Behring, Takeda, Bayer and Novo Nordisk. All fees are donated to an institutional charitable body which supports education in Haemostasis and Thrombosis. P.L.T. is a full‐time employee of Baxalta Innovations GmbH, a member of the Takeda group of companies and shareholder of Takeda Pharmaceutical Company Limited. J.S.O'D has served on the speaker's bureau for Baxter, Bayer, Novo Nordisk, Sobi, Boehringer Ingelheim, Leo Pharma, Takeda and Octapharma. He has also served on the advisory boards of Baxter, Sobi, Bayer, Octapharma, CSL Behring, Daiichi Sankyo, Boehringer Ingelheim, Takeda and Pfizer. J.S.O.D has also received research grant funding awards from 3 M, Baxter, Bayer, Pfizer, Shire, Takeda, 3 M and Novo Nordisk. J.G. has received research funding from Takeda and Roche. M.L. has served on an advisory board for CSL Behring, as a consultant for Sobi, CSL Behring and Band Therapeutics, received speaker fees from Sobi and Takeda and research funding from Takeda

## Data Availability

The data that support the findings of this study are available on request from the corresponding author. The data are not publicly available due to privacy or ethical restrictions.
